# Assessing the Effectiveness of Interactive Robot-Assisted Virtual Health Coaching for Health Literacy and Disease Knowledge of Patients with Chronic Kidney Disease: Quasiexperimental Study

**DOI:** 10.2196/68072

**Published:** 2025-01-09

**Authors:** Nai-Jung Chen, Ching-Hao Chang, Chiu-Mieh Huang, Fen-He Lin, Li-Ting Lu, Kuan-Yi Liu, Chih-Lin Lai, Chin-Yao Lin, Yi-Chou Hou, Jong-Long Guo

**Affiliations:** 1 Department of Health Promotion and Health Education College of Education National Taiwan Normal University Taipei Taiwan; 2 Department of Nursing Taiwan Adventist Hospital Taipei Taiwan; 3 College of Nursing Institute of Clinical Nursing National Yang Ming Chiao Tung University Taipei Taiwan; 4 School of Nursing College of Medicine National Taiwan University Taipei Taiwan; 5 Department of Nursing University of Kang Ning Taipei Taiwan; 6 Nephrology and Hemodialysis Center Cardinal Tien Hospital Taipei Taiwan; 7 Department of Internal Medicine Taiwan Adventist Hospital Taipei Taiwan; 8 Department of Internal Medicine Cardinal Tien Hospital Taipei Taiwan; 9 Department of Internal Medicine Fu Jen Catholic University Taipei Taiwan

**Keywords:** chronic kidney disease, disease knowledge, eHealth, health coaching, health education, health literacy, interactive robot

## Abstract

**Background:**

Chronic kidney disease (CKD) imposes a significant global health and economic burden, impacting millions globally. Despite its high prevalence, public awareness and understanding of CKD remain limited, leading to delayed diagnosis and suboptimal management. Traditional patient education methods, such as 1-on-1 verbal instruction or printed brochures, are often insufficient, especially considering the shortage of nursing staff. Technology-assisted education presents a promising and standardized solution, emphasizing the need for innovative and scalable approaches to improve CKD-specific knowledge and health literacy.

**Objective:**

This study aimed to develop and evaluate the effectiveness of an innovative 12-unit virtual health coaching program delivered through interactive robots that is intended to enhance disease knowledge and health literacy among patients with CKD.

**Methods:**

A quasiexperimental design was used, and 60 participants were evenly assigned to experimental and comparison groups. However, due to attrition, 14 participants in the experimental group and 16 participants in the comparison group completed the study. The intervention involved a 12-unit program, with each unit lasting approximately 20 minutes to 30 minutes and delivered across 3 to 4 learning sessions, and participants completed 3 to 4 units per session. The program addressed key aspects of CKD-specific health literacy including functional, communicative, and critical literacy and CKD-specific knowledge including basic knowledge, prevention, lifestyle, dietary intake, and medication. Data were collected through validated pre and postintervention questionnaires. All 30 participants completed the program and subsequent evaluations, with outcome measures assessing changes in CKD-specific knowledge and health literacy.

**Results:**

Postintervention analysis using generalized estimating equations, adjusted for age, revealed that the experimental group (n=14) had significantly greater improvements in health literacy (coefficient=2.51, Wald χ²_1_=5.89; *P*=.02) and disease knowledge (coefficient=1.66, Wald χ²_1_=11.75; *P*=.001) than the comparison group (n=16). Postintervention *t* tests revealed significant improvements in CKD-specific health literacy and disease knowledge (*P*<.001) between the experimental and comparison groups. Additional analyses identified significant group × time interactions, indicating improvements in communicative literacy (*P*=.01) and critical literacy (*P*=.02), while no significant changes were observed in functional literacy. Regarding disease knowledge, the experimental group demonstrated a significant improvement in medication (*P*<.001), whereas changes in basic knowledge, prevention, lifestyle, and dietary intake were not significant.

**Conclusions:**

This study demonstrated that interactive robot-assisted eHealth coaching effectively enhanced CKD-specific disease knowledge and health literacy. Despite the challenges posed by the COVID-19 pandemic, which constrained sample sizes, the findings indicate that this program is a promising patient education tool in clinical nephrology. Future research should involve larger sample sizes to enhance generalizability and examine additional factors influencing effectiveness.

## Introduction

Chronic kidney disease (CKD) has emerged as a major global health issue, with current estimates indicating that it affects approximately 10% of the global population [1]. The disease imposes a significant financial burden driven by the high costs of diagnosis, treatment, and ongoing care. As CKD advances, over two-thirds of patients with CKD experience complications such as cardiovascular disease, metabolic bone disorders, anemia, and malnutrition, all of which can profoundly affect their health and quality of life [2]. In Taiwan, CKD accounts for 7.14% of the nation’s total health care expenditure [3], underscoring the critical need for effective prevention strategies. The prevalence of dialysis among patients with CKD in Taiwan underscores the need for targeted interventions. In 2021, only 3.7% of dialysis patients were aged younger than 40 years, while 40.1% were aged 40 years to 64 years, 31.1% were aged 65 years to 74 years, and 25.1% were 75 years old or older. This distribution highlights that the majority of patients with CKD in Taiwan are middle-aged or older.

Health literacy (HL) is defined as the ability to access, understand, evaluate, and apply health information to make informed health decisions [4]. In this study, HL refers to a patient’s capacity to comprehend and use CKD management information to manage their CKD [5]. According to Nutbeam [6], HL consists of 3 components: functional, communicative and interactive, and critical literacy. Functional literacy encompasses the basic reading and writing skills needed for everyday functioning. Communicative literacy includes more advanced skills that enable individuals to actively engage in social contexts, extract information, and adapt to changing environments. Critical literacy involves using advanced cognitive and social skills to critically analyze information and exert greater control over personal health and life situations. Limited HL in patients with CKD has been linked to poor health outcomes and reduced treatment adherence [7]. CKD-specific HL and knowledge can help patients manage their condition effectively. In this study, disease-specific knowledge encompassed an understanding of kidney function, disease prevention, healthy lifestyle habits, diet, and medication. Higher HL levels have been identified as strong predictors of adherence to medication, diet, fluid intake, and general self-management practices [8]. A previous study suggested that complications related to kidney disease may stem from insufficient health knowledge and that HL significantly influences self-care behaviors [9]. One review highlighted that disease-specific knowledge is critical for HL, which is a prerequisite for the effective self-management of CKD [8]. Improving HL can enhance disease knowledge, promote better self-care behaviors, and potentially slow disease progression. These findings underscore the importance of HL and knowledge of kidney disease management.

Health coaching is an evidence-based approach that enables patients to make lasting behavioral changes that enhance their overall health [10]. By offering health care options tailored to individuals’ lifestyles and abilities, health coaching improves outcomes, especially among those with chronic illnesses or those at a higher risk of medication nonadherence. Previous studies have shown that health coaching can lead to positive behavioral changes and improved health outcomes in patients with chronic conditions, underscoring its value in managing long-term health [11,12].

Previous studies have demonstrated the significant impact of health promotion and education programs on patients with CKD [13]. Recent digital health intervention advancements, such as wearable devices, virtual reality, and mobile health apps, have introduced innovative approaches to enhance patient education. Wearable devices facilitate self-management by providing personalized information [14], virtual reality enhances CKD education through experiential learning [15], and mobile apps can improve disease knowledge when compared with traditional education alone [16]. Digital technologies for health coaching offer older adults support for improving various health determinants [17], and approaches such as e-learning and remote monitoring have been shown to boost treatment effectiveness and quality of life [18]. Moreover, research has also underscored the need for digital coaching to be user-friendly, highly engaging, and tailored to individual needs [19,20]. Health care professionals face challenges due to insufficient nursing staff, and CKD is a complex condition requiring comprehensive education. Most education systems rely on traditional methods (eg, brochures or 1-on-1 verbal counseling) that often lack diverse directions and interactions. This gap highlights the urgent need for innovative digital solutions to enhance CKD-specific HL and disease knowledge. Using emerging technologies (eg, interactive robots) that follow official health education guidelines [21] offers a novel solution to standardize education, address staffing shortages, and improve the overall interactivity of CKD education. These technologies can provide personalized and accessible learning experiences, as well as improve patient engagement and outcomes [22].

This study aimed to evaluate the effectiveness of an interactive robot-assisted virtual health coaching intervention for improving CKD-specific HL and disease knowledge. The following research questions guided the study: (1) How comprehensively does this intervention improve CKD-specific knowledge? (2) How effective is the intervention in enhancing CKD patients’ HL? Prototypes were developed using insights from nephrologists and nurse practitioners who served as virtual health coaches and incorporated their clinical interactions with patients. By offering an engaging and accessible educational platform, the intervention aimed to enhance patient engagement and ultimately improve the health outcomes of patients with CKD.

## Methods

### Study Design

**Figure 1:**
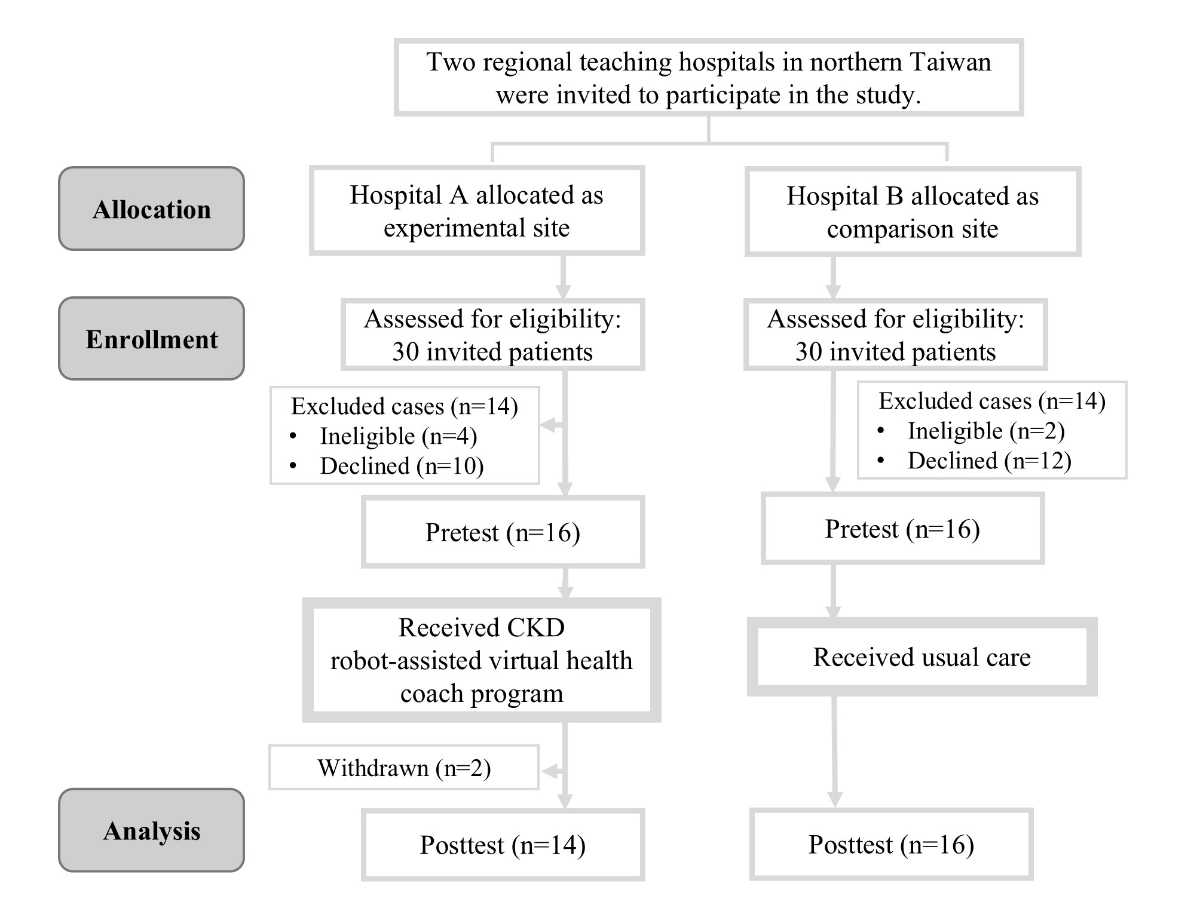
Flowchart of the study. CKD: chronic kidney disease.

This study adopted a quasiexperimental design involving the recruitment of patients with CKD from the nephrology departments of 2 regional hospitals in northern Taiwan that are similar in size and scope. Each hospital was assigned to either an experimental or comparison group. Health care professionals initially screened patients based on the eligibility criteria and referred interested parties to the research team. This process continued until 30 participants were selected from each hospital. However, due to the COVID-19 pandemic, only 14 participants in the experimental group and 16 participants in the comparison group completed the study. Participant attrition was primarily attributed to the lengthy intervention period and pandemic-related disruptions. Participants in the experimental group received both intervention and usual care provided by nurse practitioners, whereas those in the comparison group received only usual care. During recruitment, the researchers explained the use of the interactive robot and the basic interface operations required. After obtaining written informed consent, the participants were provided with an operation manual and guidance regarding the use of the interface. The flowchart of this study is presented in Figure 1.

### Study Population

The inclusion criteria required participants to be adults aged 20 years or older with a diagnosis of CKD. The exclusion criteria included dialysis, transplantation, severe mental illness, dementia, or significant sensory impairments. Patients undergoing dialysis were excluded because of significant differences in care standards and intensity compared with CKD management [23].

### Intervention

**Figure 2:**
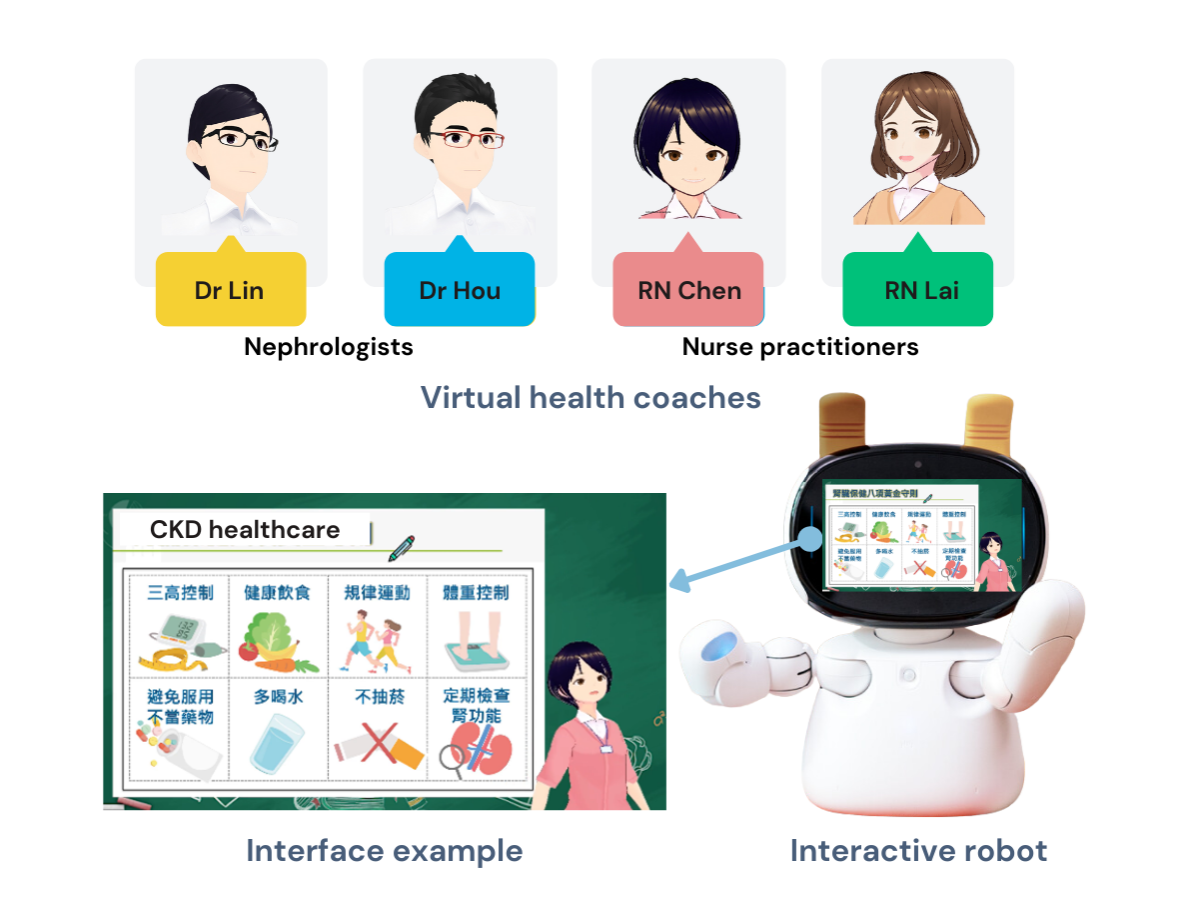
Interface example of the interactive robot-assisted virtual health coach program for chronic kidney disease (CKD). RN: registered nurse.

An interdisciplinary team including 2 nephrologists and nurse practitioners developed a 12-unit health coaching program using the interactive robot Kebbi Air S (NUWA Robotics Corp). The Kebbi robot, known for its versatility and interactivity, has been used across various domains, including education, health care, and customer service [24]. Prior studies have investigated its effectiveness for language learning and social assistance in health-related contexts and the impact of its expressions on participant engagement [25-27]. The program was designed using government-developed guidelines to ensure standardization and validity [21] after incorporating clinical experiences, patient interactions, and insights from 14 development meetings. These physicians and nurse practitioners acted as virtual health coaches through the robot, delivering content through dialogue, animations, and interactive tasks using artificial intelligence and natural language processing to facilitate dynamic patient engagement. The intervention was conducted using participant-staff 1-on-1 sessions during which staff guided participants in familiarizing themselves with the interface and provided assistance as needed. The robot was programmed to emulate health care professionals’ tone and demeanor, offering positive reinforcement (praise, encouragement, and motivational gestures) to sustain patient engagement. Correct answers on standardized quizzes were rewarded with applause, while supportive hints and explanations were provided after incorrect responses to facilitate learning. Virtual health coaches presented information tailored to the 5 stages of CKD, responded to patient inputs, and provided feedback. The program covered critical areas of CKD-specific HL, including functional, communicative, and critical literacy as well as CKD-specific knowledge, including basic knowledge, prevention, lifestyle, dietary intake, and medication. Table 1 provides an overview of the course learning objectives and corresponding outcome variables. Figure 2 illustrates an example of the program’s interface.

**Table 1:** Learning objectives and outcome variables of the intervention program.

Unit	Learning objectives	Outcome variables
1	Understand the importance of CKD^a^ self-management Know how to measure waist circumference	HL^b^: functional literacy Knowledge: lifestyle
2	Recognize CKD clinical symptoms Identify 5 warning signs of CKD	HL: critical literacy Knowledge: basic knowledge, prevention
3	Understand the importance of regular tracking Know 3 basic types of renal tests	HL: functional literacy Knowledge: prevention
4	Understand the 5 stages of CKD care Know how to calculate eGFR^c^ Learn the self-test for renal function	HL: functional literacy Knowledge: lifestyle, prevention
5, 6, 7	Understand nutrition for CKD Identify high-potassium fruits and cooking methods	HL: communicative, functional literacy Knowledge: dietary intake
8	Understand the management of comorbidities of CKD Learn about health care for CKD comorbidities	HL: critical literacy Knowledge: basic knowledge, medication
9	Understand symptoms and self-management of chronic renal failure Learn about psychological adaptations to CKD	HL: communicative literacy Knowledge: basic knowledge, medication
10	Understand the proper time for dialysis Learn the patient-physician shared decision-making strategies	HL: communicative literacy Knowledge: prevention, medication
11	Understand hemodialysis access methods Know arteriovenous fistulas and grafts	HL: critical literacy Knowledge: medication
12	Identify the emergency medical primetime^d^ Learn how to rebuild life after CKD	HL: critical literacy Knowledge: lifestyle, medication

^a^CKD: chronic kidney disease.

^b^HL: health literacy.

^c^eGFR: estimated glomerular filtration rate.

^d^The “Golden Hour” or critical period of time between the time a medical service (such as an ambulance or emergency service) receives a call for help and the arrival of the first emergency team at the scene of an emergency.

### Procedure and Program Delivery

The research team selected an experimental hospital and collaborated with attending nephrologists to define the goals, procedures, and protocols of the study. Upon receiving the hospitals’ approval to conduct the study, recruitment posters were distributed to encourage patients with CKD to participate, while an orientation session introduced participants to the study’s objectives and collaborative framework. Data collection involved 1-on-1 interviews, enabling in-depth interactions and ensuring that participants were fully engaged with the intervention. Patients were encouraged to interact with the robots to become familiar with the interface. The experimental group participated in a 12-unit intervention, with each unit lasting approximately 20 minutes to 30 minutes. Participants completed 3 to 4 units per session across 3 to 4 learning sessions. The comparison group received usual care, including personalized education, from nurse practitioners. Structured questionnaires were used for the pre and postintervention assessments.

### Measurements

The effectiveness of the intervention was assessed using a validated questionnaire [5,28]. The CKD-specific HL scale and CKD-specific disease knowledge scale were adapted from validated tools specifically designed to assess HL and disease knowledge in patients with CKD. The CKD-specific HL scale includes 17 items for functional, communicative, and critical literacy in a multiple-choice test format. The CKD-specific disease knowledge scale has 13 true or false items across 5 domains. Higher scores indicated better HL and knowledge. The Cronbach coefficients were 0.78 and 0.76 for the CKD-specific HL scale and the CKD-specific disease knowledge scale, respectively.

### Data Analysis

Data were analyzed using SPSS version 23.0 (IBM Corp). This study primarily aimed to evaluate the intervention’s effectiveness at enhancing CKD-specific HL and disease knowledge. No significant differences were found after comparing the pretest scores of the outcome variables between the experimental and comparison groups using *t* tests, eliminating the need to control for the pretest scores. However, due to missing data for some variables, generalized estimating equations (GEEs) were used to analyze the pre and posttest results between the 2 groups while also controlling for age differences. The GEE approach was selected because it effectively handles repeated measures and correlated data, making it well-suited for our study design. It is robust and accommodates missing values, providing reliable estimates and enhancing the validity of our findings.

### Ethical Considerations

This study was approved by the Joint Institutional Review Board of Shin Kong Wu Ho-Su Memorial Hospital (approval number 20211202R) and Cardinal Tien Hospital (approval number CTH-110-3-5-002). All participants were fully informed and provided their consent before the interviews began.

## Results

### User Demographics

The demographic characteristics of participants in the experimental (n=14) and comparison (n=16) groups were not significantly different in gender (χ²_28_=0.27; *P*=.61), marital status (χ²_28_=0.05; *P*=.82), and educational level (χ²_28_=0.20; *P*=.65), although the experimental group was significantly younger.

### Evaluation Outcomes

**Figure 3:**
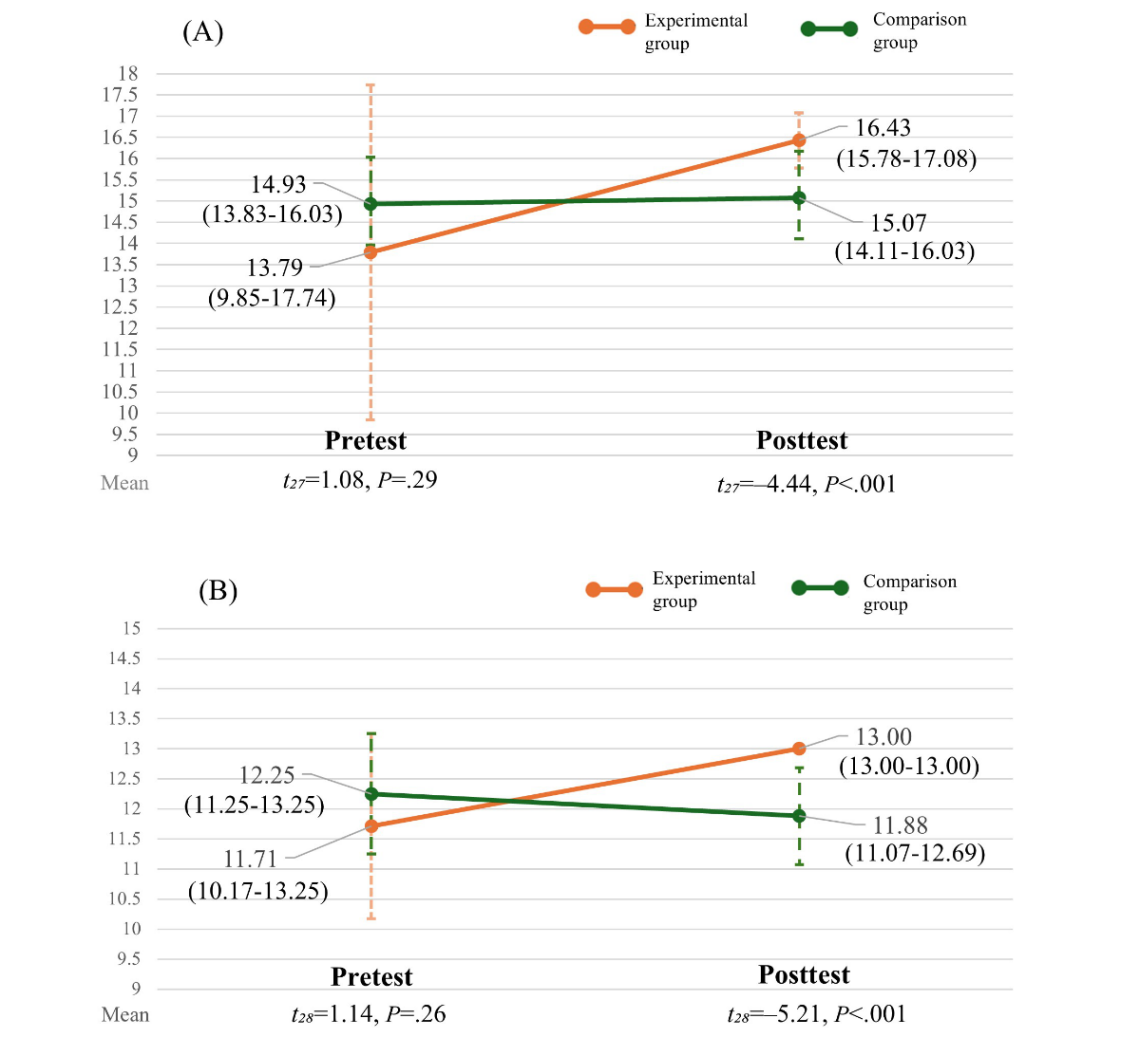
Comparison of pre and posttest (A) CKD-specific health literacy and (B) CKD-specific disease knowledge, reported as the mean (95% CI) scores, with comparisons of pre and postintervention scores between the groups.

As shown in Figure 3, the results of the *t* tests indicated that the posttest scores for CKD-specific HL (t_27_=–4.44; *P*<.001) and disease knowledge (t_28_=5.58; *P*<.001) were significantly higher in the experimental group than in the comparison group.

Table 2 shows how both groups’ results changed over time. After age adjustment, the GEE analyses demonstrated significant improvements in CKD-specific HL (*P*=.02) and disease knowledge (*P*=.001) in the experimental group compared with the comparison group. A significant group ×time interaction was observed for both CKD-specific HL and disease knowledge. Specifically, the experimental group showed improvements in HL (coefficient=2.51, Wald χ^2^_1_=5.89; *P*=.02) and disease knowledge (coefficient=1.66, Wald χ^2^_1_=11.75; *P*=.001).

**Table 2:** Results of the generalized estimating equation (GEE) analyses for outcome variables.

Variable					Coefficient (β)			SE			Wald χ^2^ (*df*)			*P* value
**CKD^a^-specific health literacy (0-17 points)**														
	Group (experimental group)^b^			–1.15			1.05			1.19 (1)			.28	
	Time (posttest)^c^			0.13			0.23			0.34 (1)			.56	
	Group (experimental) × time (posttest)^d^			2.51			1.03			5.89 (1)			.02	
	**Communicative literacy (0-7 points)**													
		Group (experimental group)^b^	–0.57			0.48			1.43 (1)			.23		
		Time (posttest)^c^	0.31			0.28			1.29 (1)			.26		
		Group (experimental) × time (posttest)^d^	1.26			0.51			6.17 (1)			.01		
	**Functional literacy (0-5 points)**													
		Group (experimental group)^b^	–0.08			0.42			0.04 (1)			.85		
		Time (posttest)^c^	–0.25			0.21			1.46 (1)			.23		
		Group (experimental) × time (posttest)^d^	0.39			0.45			0.75 (1)			.39		
	**Critical literacy (0-5 points)**													
		Group (experimental group)^b^	–0.50			0.39			1.65 (1)			.20		
		Time (posttest)^c^	–0.06			0.26			0.06 (1)			.81		
		Group (experimental) × time (posttest)^d^	0.99			0.42			5.68 (1)			.02		
**CKD-specific disease knowledge (0-13 points)**														
	Group (experimental group)^b^			–0.54			0.47			1.33 (1)			.25	
	Time (posttest)^c^			–0.38			0.28			1.82 (1)			.18	
	Group (experimental) × time (posttest)^d^			1.66			0.48			11.76 (1)			.001	
	**Basic knowledge (0-3 points)**													
		Group (experimental group)^b^	–0.30			0.23			1.75 (1)			.19		
		Time (posttest)^c^	0.13			0.12			1.07 (1)			.30		
		Group (experimental) × time (posttest)^d^	0.30			0.23			1.75 (1)			.19		
	**Prevention (0-2 points)**													
		Group (experimental group)^b^	0.07			0.06			1.07 (1)			.30		
		Time (posttest)^c^	0.07			0.06			1.07 (1)			.30		
		Group (experimental) × time (posttest)^d^	–0.07			0.06			1.07 (1)			.30		
	**Lifestyle (0-4 points)**													
		Group (experimental group)^b^	–0.05			0.24			0.03 (1)			.85		
		Time (posttest)^c^	0.06			0.17			0.14 (1)			.70		
		Group (experimental) × time (posttest)^d^	0.30			0.25			1.36 (1)			.24		
	**Dietary intake (0-** **2** **points)**													
		Group (experimental group)^b^	–0.02			0.13			0.02 (1)			.89		
		Time (posttest)^c^	–0.13			0.15			0.70 (1)			.40		
		Group (experimental) × time (posttest)^d^	0.27			0.18			2.30 (1)			.13		
	**Medication (0-2 points)**													
		Group (experimental group)^b^	–0.23			0.15			2.32 (1)			.13		
		Time (posttest)^c^	–0.44			0.18			6.17 (1)			.01		
		Group (experimental) × time (posttest)^d^	0.80			0.22			13.32 (1)			<.001		

^a^CKD: chronic kidney disease.

^b^Reference group (group): comparison group.

^c^Reference group (time): pretest.

^d^Reference group (group × time): comparison group × pretest.

The group differences in patterns of change over time shown in Table 2 also demonstrated that the experimental group had significant improvements in the scores for communicative literacy (coefficient=1.26, Wald χ^2^_1_=6.17; *P*=.01) and critical literacy (coefficient=0.99, Wald χ^2^_1_=5.68, *P*=.02) compared with the comparison group, but functional literacy did not significantly improve. Regarding CKD-specific disease knowledge, the experimental group showed significant improvement in medication (coefficient=0.80, Wald χ^2^_1_=13.32; *P*<.001) compared with the comparison group, but there were no significant changes in areas such as basic knowledge, prevention, lifestyle, or dietary intake.

## Discussion

### Principal Findings

This study demonstrated preliminary evidence to support interactive robot-assisted virtual health coaching as an intervention tool for patients with CKD. Notably, participants in the experimental group showed significant improvements in both HL and disease knowledge compared with those in the comparison group. The experimental group was significantly younger than the comparison group, which may have influenced their receptiveness to the technology. However, prior studies indicate that older adults generally exhibit similar attitudes toward robots to those of younger individuals, challenging the stereotype of their lower robot-related receptiveness [29]. Furthermore, older adults often prefer human-like robots, such as the Kebbi robot, highlighting the importance of robot design and appearance in fostering acceptance [30,31]. These findings indicate that technology-enhanced educational interventions can effectively enhance HL and disease knowledge, which are associated with improved CKD-related outcomes.

The improvements observed in CKD-specific HL and disease knowledge were consistent with the benefits of technology-supported health education programs. Previous studies have shown that interactive digital interventions, particularly personalized coaching, can boost patient engagement and improve health outcomes [32,33]. This study builds on previous research by demonstrating the effectiveness of interactive robot-assisted virtual health coaching in managing chronic diseases. Our multidisciplinary approach included health professionals such as nephrologists and nurse practitioners, whose clinical experiences were integrated into the course content and represented by virtual health coaches [34]. Previous research has shown that avatar-based education has a positive effect on patient engagement and HL, which aligns with our findings [35].

A literature review of digital health coaching for older workers highlighted how virtual health can support healthy aging [36]. Although said review focused on a different group, it showed that digital interventions could effectively enhance health knowledge and well-being, especially when adopting a user-centered approach. This aligns with the findings of our study, which designed the intervention using health care professionals’ clinical experiences with patients. By integrating these real-world interactions, the digital tools were tailored to address the practical needs and circumstances of patients with CKD, making the content more relevant and applicable. These results underscore the importance of health care professionals’ insights when developing effective digital health interventions.

Participants in the experimental group showed significant improvements in communicative and critical literacy following the intervention, whereas functional literacy was not significantly changed. This pattern is consistent with the findings of a previous study in the Netherlands, which found that communicative HL and critical HL were more influential on chronic disease self-management, whereas functional literacy was less impactful [37]. Furthermore, a recent cross-national study involving geriatricians from Europe and Japan highlighted that virtual health interventions could enhance HL, emotional well-being, and social engagement among older adults, emphasizing the benefits of personalized digital coaching [38]. Our findings suggest that interactive virtual health coaching successfully supported patients in comprehending and using health information, making informed decisions, and critically evaluating health messages. However, the lack of improvement in functional literacy may be attributed to the program’s emphasis on enhancing communication, lifestyle modifications, and decision-making skills rather than basic skills such as reading medical documents or interpreting health indicators. Additionally, certain robot interface-related limitations may have impeded the delivery of more comprehensive health information necessary for developing functional skills. Future iterations should include more comprehensive content on functional skills to address all aspects of HL, thereby enhancing its overall effectiveness.

In addition to literacy, this study evaluated the effectiveness of the intervention on CKD-specific knowledge. The results showed a significant improvement in medication use in the experimental group, whereas no notable changes were observed in other areas such as basic knowledge, prevention, lifestyle, or dietary intake. This suggests that the program’s structured content was particularly effective for teaching patients about medication. The materials, designed by health care professionals based on their clinical experiences, incorporated visual aids and interactive discussions, making the content more engaging and easier to understand. This aligns with the findings of Occa et al [39], who highlighted that interactive features and visual informativeness help improve patient knowledge by enhancing cognitive absorption. Similarly, Hassan and Davies [40] found that virtual health tools improve medication adherence by boosting patient HL and reducing barriers, emphasizing convenience and empowerment. The limited improvement in other CKD-related knowledge areas may have been due to the small sample size, which may have affected the statistical significance of our findings. Additionally, some participants reported that similar topics had already been covered in educational brochures, which might have reduced their motivation to engage fully. A more comprehensive approach that places greater emphasis on prevention, lifestyle changes, and dietary guidance is required to address these areas better.

### Implications

This study has important implications for integrating digital health technologies into CKD education. Interactive robots, combined with a virtual health coaching program, have exhibited promise in enhancing HL and disease knowledge, particularly in resource-limited settings where health care resources are scarce and chronic disease management is challenging. By leveraging health care professionals’ expertise and adhering to official health education guidelines, this study emphasizes the importance of creating standardized and engaging digital education tools that effectively meet patient education requirements. Furthermore, prior studies have demonstrated the potential of robots as impactful didactic tools in health science education, yielding promising results for enhancing cognitive therapies and facilitating health care intervention [41,42]. Expanding the use of such innovations may contribute to more effective patient education, improved disease knowledge, and enhanced HL, ultimately leading to improved CKD management.

### Limitations

Despite these promising findings, this study had several limitations. First, participants in the experimental group were significantly younger than those in the comparison group, partly due to recruitment challenges during the COVID-19 pandemic, which hindered the enrollment of a sufficient number of older participants. Although age differences were controlled in the GEE analysis, which helped mitigate their impact, they remain a potential source of bias. Future research should further explore age-related differences, potentially incorporating multivariable analyses, to better understand and minimize their effects, ensuring the findings’ broad applicability. This study primarily aimed to evaluate intervention effectiveness. Though digital literacy gaps related to age differences were not the primary focus, they represent an important area for future research.

The relatively small sample size limited the findings’ generalizability, and 1-on-1 recruitment made larger sampling challenging. Statistical power was not explicitly considered during the study’s design due to the lack of feasibility data, which accounts for the absence of a formal power calculation. Future studies should prioritize power analysis and incorporate larger sample sizes to confirm these results and enhance external validity. Additionally, the quasiexperimental design limited our ability to draw causal conclusions, suggesting that future research should use randomized controlled trials to strengthen causal inferences.

Another limitation involved the Taiwanese CKD population’s unique characteristics, including dietary habits, medication use (eg, traditional Chinese medicine), and lifestyle factors, which may limit the findings’ applicability to other populations. These cultural differences introduce potential biases, and future research should consider these factors to improve the results’ generalizability.

### Conclusions

This study demonstrated that interactive robot-assisted virtual health coaching could potentially enhance CKD management by improving disease knowledge and HL. To our knowledge, this is the first study to use an interactive robot for CKD health education, presenting a novel approach that alleviates the burden on health care professionals. Developed by a multidisciplinary team, the program delivers standardized, credible education while engaging patients in their care. The findings emphasize the scalability of digital health solutions, particularly in resource-limited settings, offering a valuable tool for nephrology practices. Future research should validate these results across diverse populations, examine the long-term effects on HL and disease outcomes, and refine the intervention based on patient feedback to optimize its content and usability. Integrating digital tools into clinical practice could significantly advance patient education and chronic disease management.

## Abbreviations

CKD: chronic kidney disease
GEE: generalized estimating equations
HL: health literacy

## Data Availability

The data sets generated or analyzed in this study are available from the corresponding author upon reasonable request.
